# Effect of Alumina Incorporation on the Surface Mineralization and Degradation of a Bioactive Glass (CaO-MgO-SiO_2_-Na_2_O-P_2_O_5_-CaF_2_)-Glycerol Paste

**DOI:** 10.3390/ma10111324

**Published:** 2017-11-18

**Authors:** Dilshat Tulyaganov, Khasan Abdukayumov, Olim Ruzimuradov, Mirabbos Hojamberdiev, Emanuel Ionescu, Ralf Riedel

**Affiliations:** 1 Turin Polytechnic University in Tashkent, 17, Niyazova 100095, Uzbekistan; tulyaganovdilshat@gmail.com (D.T.); hasaniy04@gmail.com (K.A.); ruzimuradov@rambler.ru (O.R.); hmirabbos@gmail.com (M.H.); 2 Institute for Materials Science, Technische Universität Darmstadt, Jovanka-Bontschits-Strasse 2, D-64287 Darmstadt, Germany; riedel@materials.tu-darmstadt.de

**Keywords:** bioactive glass, bioactive glass pastes, organic carriers, bioactivity, hydroxyapatite (HA), HA mineralization

## Abstract

This study investigates the dissolution behavior as well as the surface biomineralization in simulated body fluid (SBF) of a paste composed of glycerol (gly) and a bioactive glass in the system CaO-MgO-SiO_2_-Na_2_O-P_2_O_5_-CaF_2_ (BG). The synthesis of the bioactive glass in an alumina crucible has been shown to significantly affect its bioactivity due to the incorporation of aluminum (ca. 1.3–1.4 wt %) into the glass network. Thus, the kinetics of the hydroxyapatite (HA) mineralization on the glass prepared in the alumina crucible was found to be slower than that reported for the same glass composition prepared in a Pt crucible. It is considered that the synthesis conditions lead to the incorporation of small amount of aluminum into the BG network and thus delay the HA mineralization. Interestingly, the BG-gly paste was shown to have significantly higher bioactivity than that of the as-prepared BG. Structural analysis of the paste indicate that glycerol chemically interacts with the glass surface and strongly alter the glass network architecture, thus generating a more depolymerized network, as well as an increased amount of silanol groups at the surface of the glass. In particular, BG-gly paste features early intermediate calcite precipitation during immersion in SBF, followed by hydroxyapatite formation after ca. seven days of SBF exposure; whereas the HA mineralization seems to be suppressed in BG, probably a consequence of the incorporation of aluminum into the glass network. The results obtained within the present study reveal the positive effect of using pastes based on bioactive glasses and organic carriers (here alcohols) which may be of interest not only due to their advantageous visco-elastic properties, but also due to the possibility of enhancing the glass bioactivity upon surface interactions with the organic carrier.

## 1. Introduction

The application of glasses as “biomaterials” has revolutionized the field of human biomedicine and has brought the concept of “surface active” materials which have the ability to elicit a special response on their surface when in contact with biological fluids [1,2]. It has been well accepted and agreed that the key feature which leads to the bone-bonding ability of bioactive glasses (and of bioactive materials in general), is the formation of hydroxycarbonate apatite (HСA) on their surface. This layer considerably enhances the interfacial adhesion of the implant to bone tissue and therefore a stable interface is maintained long enough to favor further cellular interaction such as the incorporation of collagen and interaction with other biomolecules and tissue growth factors, which then favor the development of a biological bond with the tissues [3]. Consequently, this interface requires tailor-made biomaterials with specific and adjustable chemical reactivity, considering the need to match the rates of implant dissolution and tissue growth. Typically, silicate-based bioactive glasses are able in the very early stage of mineralization to form a silica gel layer onto their surface, which is of crucial importance for their bone-bonding behavior. There has been also an alternative opinion which considers the mineralization of HСA layer on the glass surface not critical for the bioactivity of the material [4], as the ionic dissolution products from the bioactive material appear to stimulate the growth and differentiation of cells at the genetic level, an effect which has been considered to be dose dependent. Despite the controversy related to the importance of HСA layer formation on the surface of bioactive glasses/ceramics, it is still considered to be the marker of bioactivity during the initial screening of biomaterials [4].

In the last few years, efforts concerning the design and processing of bioactive materials in the system CaO-MgO-SiO_2_-Na_2_O-P_2_O_5_-CaF_2_ resulted in new series of glasses, which were shown to exhibit high bioactivity in vitro, that is, excellent biomineralization upon immersion in simulated body fluid (SBF), stimulation of osteoblast proliferation in cell culture medium, and no evidence of any toxicity or other detrimental effects in the functionality of cells [5,6,7]. Subsequent clinical trials were successfully undertaken with glass particulates that formed a cohesive mass with patient’s blood, demonstrating its homeostatic effect [8]. Furthermore, aiming at better quality of a grafting procedure, injectable pastes were produced using a melt-quenched CaO-MgO-SiO_2_-Na_2_O-P_2_O_5_-CaF_2_ bioactive glass and two organic carriers, namely polyethylene glycol (PEG) and glycerol (gly) [9]. The prepared homogeneous mixtures appeared in the form of moldable pastes and demonstrated cohesive injectability [9]. The excellent bioactivity of those pastes in vitro was expressed by high mineralization rates of crystalline hydroxyapatite (HA), which was identified by X-ray diffraction analysis (XRD) already after 12 h of immersion in simulated body fluid (SBF) [9]. More recent experimental data clearly indicated the fact that the processability and bioactivity of the pastes can be adjusted by the organic carrier. In a recent case study, it was shown the occurrence of chemical bonding between the surface of bioactive glass particulates and glycerol (gly), whereas only physical interactions were detected in the case of organic carrier based on PEG [10].

The aim of present study was to elucidate the effect of alumina incorporation on the surface mineralization and degradation of bioglass (BG) and a bioglass-glycerol (BG-gly) paste. Within this context, the effect of the synthesis of BG in an alumina crucible (unlike in other reported studies, which used a Pt crucible [5,6,7,9,10]), as well as the effect of glycerol interaction with the BG surface on the bioactivity was carefully assessed via SBF tests and extensive structural analysis of the studied materials. It is shown that the synthesis condition of BG affects its network connectivity and consequently alters its bioactivity; moreover, the BG-gly sample exhibits strong chemical interactions between gly and the BG surface, which boost the bioactivity of the paste in comparison to that of the neat BG.

## 2. Materials and Methods

### 2.1. Materials Preparation

Glass with the nominal composition (mol %) 4.33 Na_2_O-30.30 CaO-12.99 MgO-45.45 SiO_2_-2.60 P_2_O_5_-4.33 CaF_2_ was produced from following materials: powders of technical grade silicon oxide (purity 99.5%); calcium carbonate (99.5%), and; reagent grade MgCO_3_, Na_2_CO_3_, CaF_2_, and NH_4_H_2_PO_4_. Homogeneous mixtures of batches (100 g), obtained by ball milling, were preheated at 1000 °C for 1 h for decarbonization and then completely melted in alumina crucibles at 1420 °C for 1 h, in air. Glass frit was produced by quenching of the melts upon immersing them into cold water. The frit was dried, crushed and milled in a high-speed planetary mill (Nannetti, Faenza, Italy; balls/material weight ratio was approximately 2/1) and subsequently passed through a 40 µm sieve to obtain fine powders with 5–6 µm mean particle size; mean particle size, calculated by the particle size distribution curves, obtained by light scattering technique (Coulter LS 230, Fraunhofer optical model, Beckman Coulter, High Wycombe, UK). Pure glycerol for analysis was supplied by Fluka and was used to prepare the BG-gly sample.

Glass particulates (73 wt %) were premixed with glycerol (27 wt %) in plastic boxes using a metallic spatula before feeding the mixture in a laboratory mixer (IKA RW 47 Digital Pilot-Process Mixer, Cole Parmer, London, UK) at room temperature and 30 rpm. The as-obtained paste was easily transferred and stored into standard syringes.

### 2.2. In Vitro Bioactivity Tests and Materials Characterization

The apatite-forming ability of BG and BG-gly paste were investigated upon immersion in simulated body fluid (SBF) at 37 °C. The conditions were maintained in agreement to those reported in our previous study [9], keeping the sample-to-SBF ratio as 2 mg/mL. The SBF solution had following ionic concentrations: Na^+^ 142.0; K^+^ 5.0; Ca^2+^ 2.5; Mg^2+^ 1.5; Cl^−^ 147.8; HPO_4_^2−^ 1.0; HCO_3_^−^ 4.2, and; SO_4_^2−^ 0.5 mmol L^−1^; thus, its ionic composition being nearly equivalent to human plasma, as discussed by Tas [11]. It should be mentioned that the assessment of the bioactivity in the silicon oxycarbide presented here involves preliminary acellular SBF soaking studies, which have specific limitations and have been critically discussed in the literature [4,12]. The sample SBF mixtures were sealed immediately after preparation into sterilized plastic flasks and were placed in an oven at 37 °C (±0.5 °C), which was agitating in a circular motion at 120 rpm. The sampling took place at different times varying between 1 h and 7 days. The experiments were performed in duplicate in order to ensure the accuracy of results. After each experiment, the solids and liquids were separated and pH was measured. X-ray diffraction (XRD) measurements were performed with a STOE X-ray diffractometer (Stoe&Cie GmbH, Darmstadt, Germany) in transmission geometry (Mo Kαradiation). Microstructure observations were done by field emission scanning electron microscopy (JSM-7600F, JEOL, Akishima shi, Japan). Energy dispersive spectroscopy (EDS) was employed for chemical analysis. The analysis of the supernatant liquid comprised pH measurements and determination of the concentration of Ca^2+^, Mg^2+^, P^5+^, Si^4+^, Na^+^ and Al^3+^ by inductively coupled plasma optical emission spectroscopy (ICP-OES, Jobin Yvon, JY 70 plus, Longjumeau, France). The measurements were done in triplicate. Typical error for the ICP-OES measurements has been 5%. Wavelengths used to analyze the presence of the elements by ICP-OES were as follows: sodium, 589.592 nm; magnesium, 280.271 nm; calcium, 393.366 nm; phosphorus, 213.617 nm; silicon, 251.611 nm; aluminum, 396.153 nm.

### 2.3. Dissolution Tests

Dissolution tests were designed according to the standard ISO 10993-14 “Biological evaluation of medical devices—Part 14: identification and quantification of degradation products from ceramics” in the most frequently encountered in vivo pH (7.4) [8]. The dissolution behavior was investigated by immersion of the samples in freshly prepared Tris-HCl solution at 37 °C in static regime without solution replacement. The sampling was done after 120 h via filtering off the solid phase. The solid samples were washed in deionized water and dried in an oven to constant weight (i.e., samples were dried and their weight was measured in specific time intervals; samples were considered dry when the weight for two consecutive readings was constant [13]). Their weight was measured and the mass loss of the glass samples after 120 h of immersion in Tris-HCl solutions was calculated. The experiments were made in triplicate in order to assure the reproducibility of the obtained results.

## 3. Results

**Figure 1:**
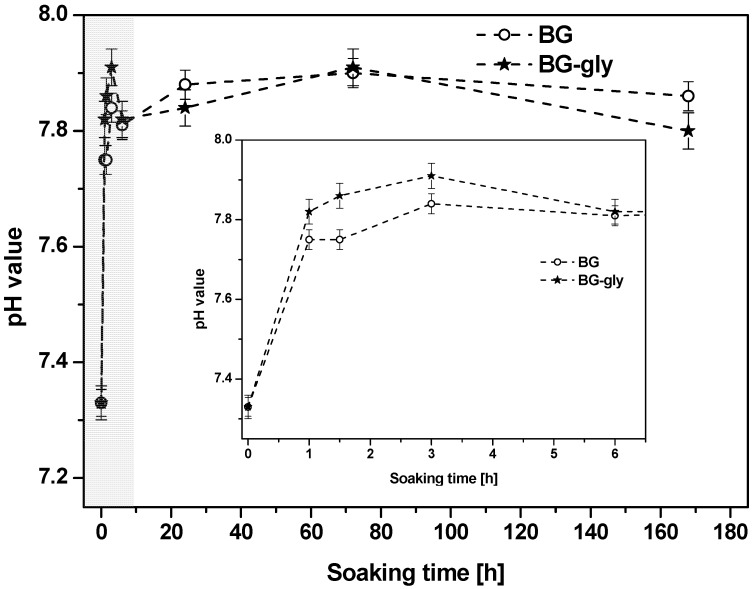
Evolution of pH value in simulated body fluid (SBF) as a function of the immersion time for bioglass (BG) and bioglass-glycerol (BG-gly) paste; the inset represents the magnification of the grayish area of the plot.

Figure 1 shows the evolution of the pH value of the SBF after testing BG and BG-gly for 1, 1.5, 3, 6, 24, 72 and 168 h. The change in pH occurs immediately after soaking the materials in SBF and is particularly pronounced within the first hour of incubation time. Thus, it seems that the dissolution takes place immediately after immersion in SBF and in all cases alkaline reaction occurred, that is, abrupt increase of pH value (Figure 1), attributed to the rapid ion exchange between M^+^/M^2+^ cations from the glass (network modifiers, i.e., M^+^ = Na^+^ and M^2+^ = Ca^2+^, Mg^2^) with hydronium ions (H_3_O^+^) from the solution. Subsequently, a steady increase of the pH value is observed over 3 days of soaking in SBF. In this period, namely, between 1 and 3 h of SBF exposure, the pH of the solution of BG-gly appears to be slightly higher than that of BG, whereas no significant difference between BG and BG-gly is observed at longer exposure times. The pH change correlates to the evolution of the ionic concentrations of Ca^2+^, Mg^2+^, P^5+^, Si^4+^, Na^+^ and Al^3+^ cations in the liquid.

**Figure 2:**
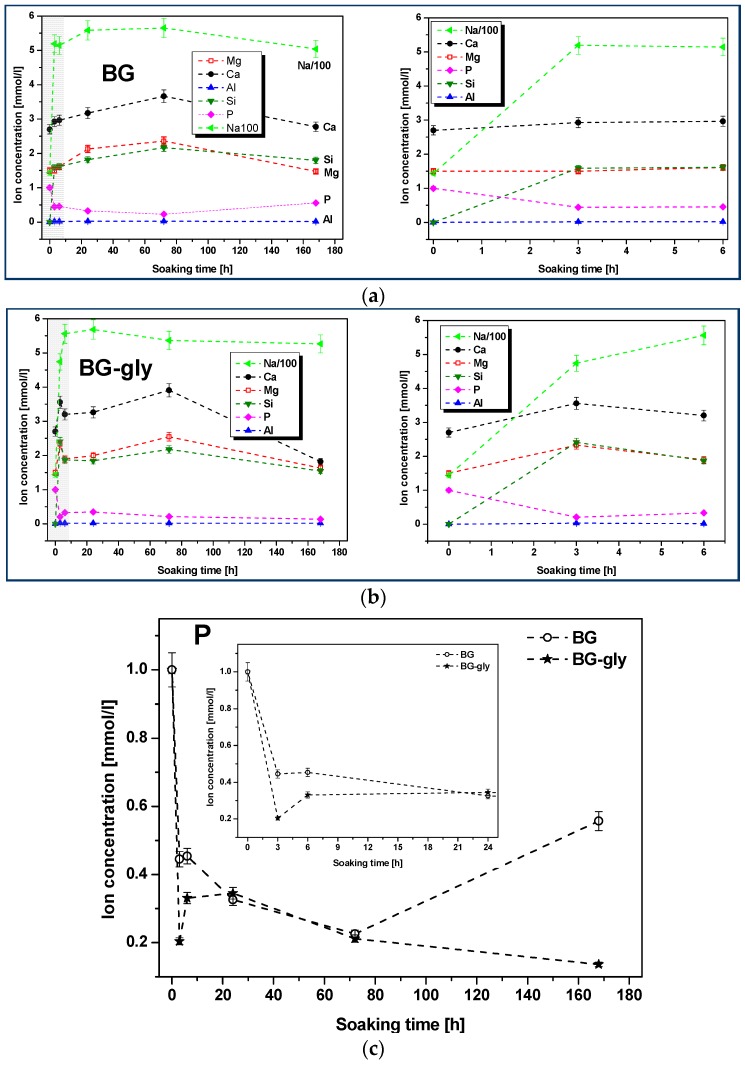

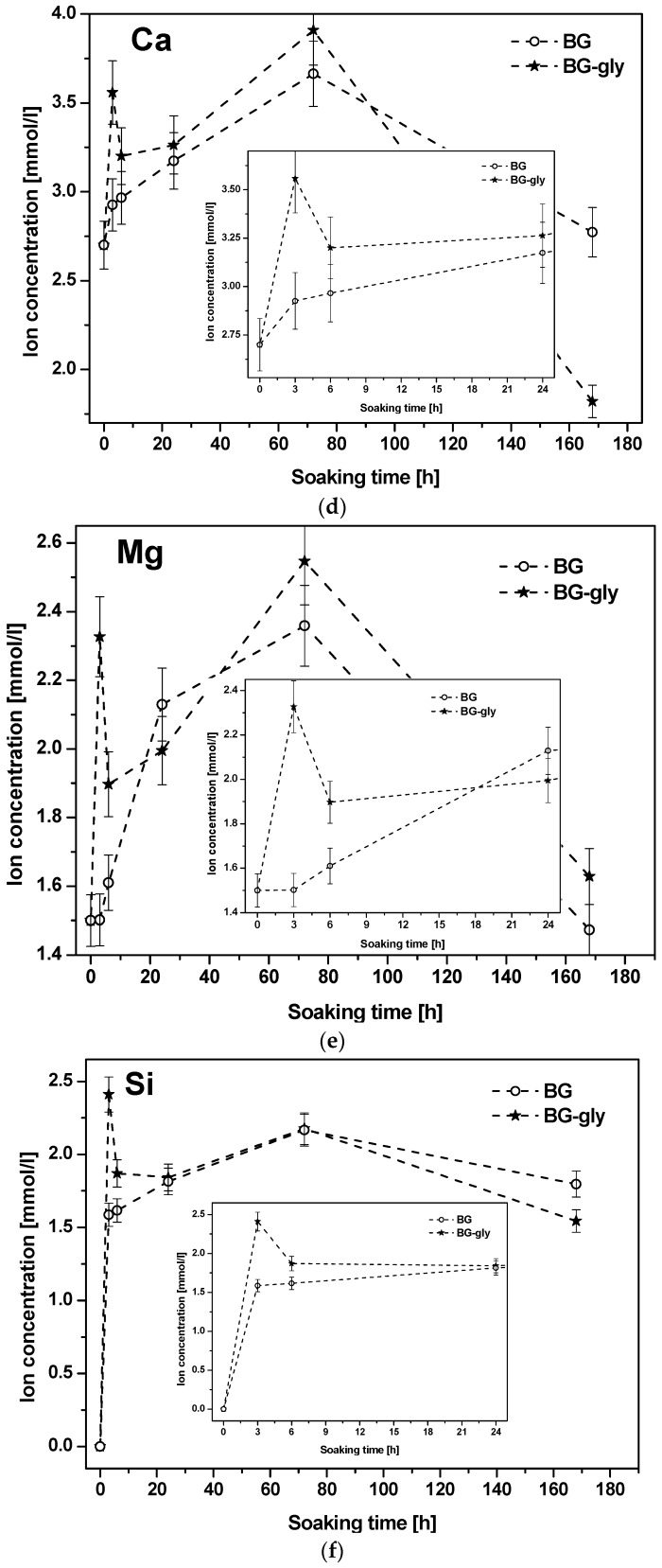
Ionic concentrations in SBF as function of immersion time in SBF: (**a**) BG; (**b**) BG-gly paste (right plots represent magnifications of the grayish areas from the left plots, respectively); (**c**–**f**) comparison of the evolution of selected ionic concentrations for BG and BG-gly (insets represent magnifications of the soaking time range up to 24 h).

Figure 2a,b shows the plots of the molar concentrations of the mentioned species as functions of the SBF immersion time for BG and BG-gly. It can be observed that the increase of the concentration of the Na^+^, Ca^2+^ and Mg^2+^ cations is steep in the early stage of the immersion. Subsequently, the concentration of Na^+^, Ca^2+^ and Mg^2+^ continuously increased until 72 h immersion in SBF. It is worth mentioning that, although the concentrations of the alkaline/alkaline-earth ions leached from BG and BG-gly into the SBF solution are comparable with each other, the concentrations of Ca^2+^ and Mg^2^ are quite different. Thus, at the initial stages (e.g., after 3 h) of exposure to SBF, the BG-gly paste featured considerably higher Ca^2+^ and Mg^+2^ concentrations as compared to the neat BG sample, that is, ca. 25 and 50% higher concentrations, respectively (Figure 2d,e). The phosphate concentration rapidly decreased in the early stage of the immersion and again, the concentration drop was more pronounced in the case of BG-gly than for the neat BG, indicating the active role of phosphate on the transformation of its surface (Figure 2c). The concentration of Al^3+^ was very low in the SBF solution for both BG and BG-gly, being at the limit of the sensitivity of the ICP-OES apparatus.

**Figure 3:**
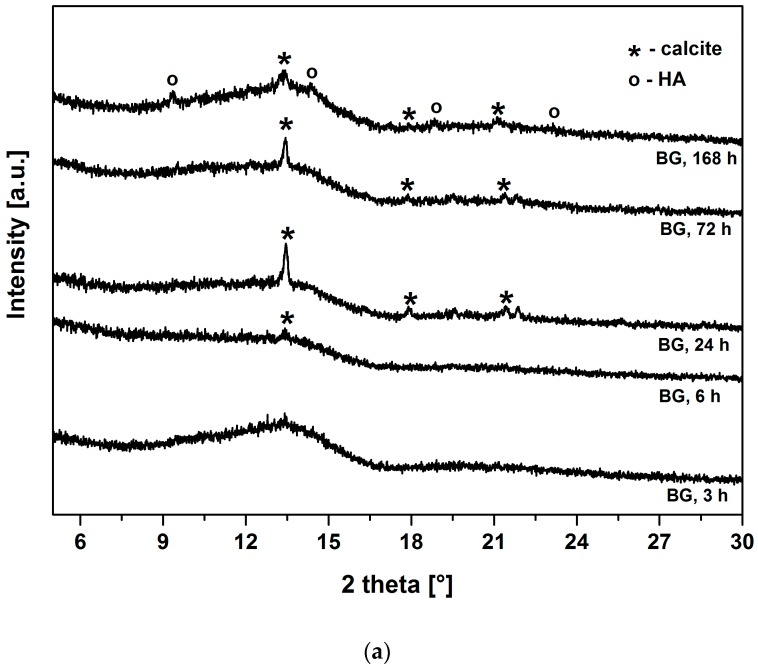

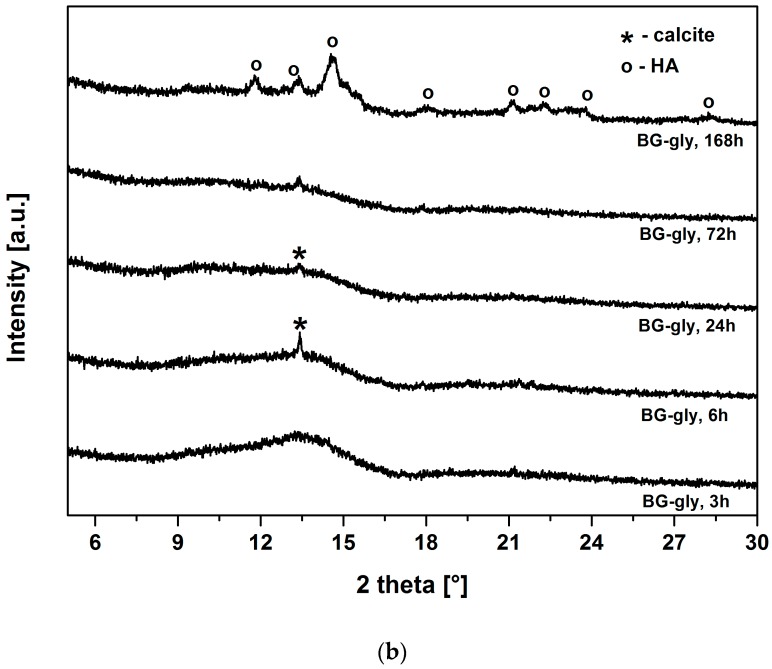
X-ray diffraction (XRD) patterns of (**a**) BG; (**b**) BG-gly paste after immersion in SBF for different periods of time.

The difference in leaching behavior of BG and BG-gly, which has a crucial importance for the mineralization of HA, is likely governed by the surface chemistry, which was assessed by means of XRD and SEM. Figure 3a,b show the XRD patterns of BG and BG-gly, respectively, after immersion in SBF for various periods of time. Both BG and BG-gly are nearly featureless after 3 h of immersion; whereas calcite with its main reflection at 2θ = 13.42° was revealed as a primary crystalline phase in BG-gly after 6 h of SBF exposure (Figure 3b). In the case of BG, the reflections of calcite are clearly distinguishable already after 24 h of immersion in SBF (Figure 3a). Calcite with its characteristic reflections remains the only crystalline phase at the surface of BG after 72 h of exposure to SBF, although the intensity of the reflections decreases in time. A new reflection at 2θ = 14.57°, was revealed in BG after 168 h of soaking in SBF was assigned to HA and, thus, the surface of BG consisted of calcite and hydroxyapatite (Figure 3a). In the case of the BG-gly paste, the reflections of the calcite phase completely vanished after 168 h of soaking in SBF and HA was the only crystalline phase at the surface of the BG-gly sample (Figure 3b). 

**Figure 4:**
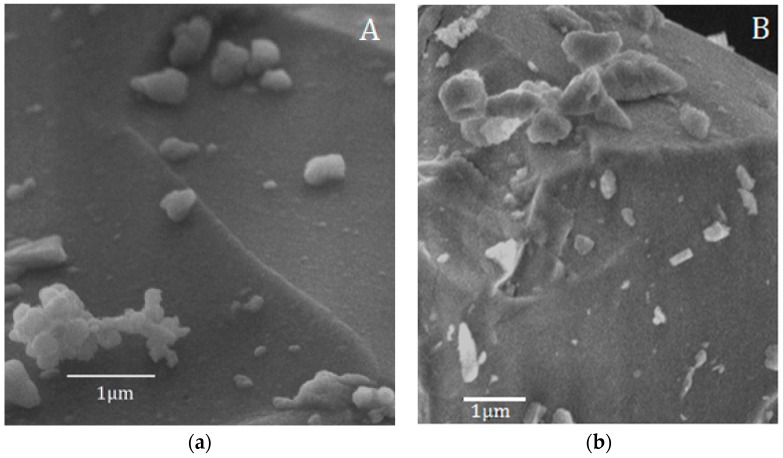
Scanning electron microscope (SEM) micrographs after immersion in SBF for 168 h (7 d): (**a**) BG; (**b**) BG-gly paste.

These results are in accordance with the SEM observation of the samples after 168 h of soaking in SBF (Figure 4), which demonstrated that the surface of the BG-gly sample features submicron precipitates enriched in Ca, P, and Si according to EDS (not shown). EDS from larger areas of the surfaces revealed the presence of Al in both BG (1.33 wt %) and BG-gly (1.37 wt %) samples, clearly evidencing that this element becomes incorporated into the glass structure during its preparation stage in an alumina crucible. This is in very good agreement with observations from Goel et al. [14], who showed that the aluminium uptake from the alumina crucibles during the glass preparation process may be as high as 1.5–2.0 wt % Al_2_O_3_ (i.e., 0.8–1.1 wt % Al). It should be noted that the ICP-OES measurements revealed negligible leaching of Al from the samples into the SBF solution. The slightly higher content of Al on the surface of BG and BG-gly (as compared to results of the study from [14]) is probably related to the leaching of the other ions into SBF solution [6].

It is well known that alumina may act as a network former or modifier, depending on the ratio between the aluminum cations and the other network modifiers. When fulfilling the function of a network former, aluminum is incorporated within the glassy network in the form of AlO_4_ tetrahedra, provided that the total concentration of alkali and/or alkaline earth oxides equals or exceeds that of alumina [15]. Consequently, each added aluminum ion is able to remove one non-bridging oxygen (NBO) from the glass structure due to (i) the lack of the oxygen provided by alumina and (ii) the necessity to maintain local charge neutrality of the AlO_4_ units. As a result, the network connectivity of the glass structure is expected to increase. Moreover, it has been accepted that the presence of Al^3+^ increases the chemical durability of glasses by forming Al–OH groups on their surface when in contact with body fluids [16]. According to Shelby et al. [15], the addition of alumina to the glass typically improves its durability in neutral solutions, but results in very rapid dissolution in highly acidic solutions due to attack at the Al–O bonds. Hench and Anderson concluded that alumina inhibits HA formation, thereby retarding the mineralization of osteoids into bone tissue [17]. The formation of Al–OH bonds on the glass surface hinders the apatite formation possibly by the following two mechanisms: (i) it decreases the concentration of silanol groups on the glass surface (which act as nucleating centers for apatite formation); (ii) the Al_2_O_3_ gel is positively charged at pH = 7.4, while the SiO_2_ gel-like layer is negatively charged [18]. Nevertheless, minor amounts of Al_2_O_3_ (0.5–2 mol %) may, for instance, improve the sintering ability of bioactive glasses and reduce their crystallization tendency. Thus, Al_2_O_3_-containing apatite-mullite glass-ceramics have been shown to be biocompatible in vivo [19], but featured the absence of HA formation on their surface due to the lack of silanol (Si–OH) groups.

Considering the behavior of BG upon SBF exposure in the present study and comparing this with the behavior of the BG prepared in a Pt crucible, we can confirm the literature known effect of Al which improves the network connectivity and consequently pushes down the HA crystallization. We consider that the small amount of Al in the BG glass strengthens the glass network and induces an enrichment of Al–OH groups at the surface of BG upon exposure to SBF, thus suppressing the HA mineralization.

The chemical degradation tests performed for BG and BG-gly in Tris-HCl revealed a considerably mass loss of 2.81 wt % for the BG sample and of 28.46 wt % for BG-gly. Considering that the BG-gly sample consists of 27 wt % glycerol (which gets dissolved upon SBF immersion), we can estimate the mass loss of BG in the BG-gly paste to ca. 1.46 wt %. This can be explained by the fact that upon immersion of BG-gly into SBF, first glycerol should get dissolved; as some glycerol is chemically bonded to the surface of BG [10], one may speculate that the chemically bonded BG might be responsible for the lower mass loss observed for the BG-gly samples. In the case that the chemically bonded glycerol also gets leached into SBF (in time), the lower mass loss of BG-gly as compared to that of BG may be considered as a consequence of the lower dissolution rate of BG in BG-gly due to the presence of the surface-bonded glycerol. However, the chemically bonded glycerol may not get released upon SBF exposure and thus the mass loss of the BG-gly sample might be even lower than 1.46 wt %. Interestingly, the pH value of the solution after immersion for 120 h in Tris-HCl increased for both investigated samples from the initial value of 7.4 to the value of 8.7.

## 4. Discussion

The mechanism of apatite formation is the subject of much scientific interest and can be described as occurring through a series of various chemical reactions. As the result of ion exchange, the glass forms Si-OH groups on its surface. Simultaneously with the ion exchange, water directly attacks the glass network (so-called congruent dissolution [15]). The release of alkali and alkaline earth ions from the glass causes/affects the crystallization kinetics of HA, while the presence of the silanol groups on the surface of the glass is of crucial importance to induce (i) heterogeneous nucleation of apatite and (ii) the release of Ca ions from the materials. As a consequence, amorphous calcium phosphates are formed on the surface of the glass and convert subsequently to crystalline HA [1,2,20].

In our previous study [9], BG melt glass was prepared in a Pt crucible and its bioactivity was studied upon SBF exposure. Both BG and the corresponding BG-gly paste featured high bioactivity in vitro and showed the formation of crystalline hydroxyapatite (HA) already after 12 h immersion in SBF. In the present study, the same BG glass prepared in alumina crucible and the corresponding BG-gly paste have been shown to contain small amounts of aluminum in their network. As a consequence, the mineralization of HA on the surface of the BG samples has been suppressed. Likewise, ZnO was reported to suppress in a similar manner the HA mineralization upon SBF exposure [21]. The reason for this behavior was considered to rely on the significant decrease of the release of silicon from the glass-ceramic as well as a lack of silanol groups that caused suppression of apatite formation on its surface. In the absence of ZnO or at low contents, the glass-ceramic was able to show HA mineralization upon SBF exposure [21].

The release of different species to solution has been known to depend on the strength of cation-oxygen bonds that must be broken to generate a detached unit that can be solvated and released into the solution. The experimental observation revealed that, for instance, sodium bonded to silicate groups is released more easily than sodium bonded to aluminate groups in aluminosilicate glasses [22]. This may be a reason of the alteration in the HA mineralization behavior of BG and BG-gly prepared in this study as compared to those based on BG prepared in Pt crucible.

The surface of both materials from the present study (BG and BG-gly) apparently is able to promote HA precipitation. The XRD results (Figure 3) indicate that calcite has been formed at the expenses of or concurrently to HA at the early stage of immersion in SBF. Calcite precipitation might occur due to depletion in PO_4_^3−^ ions followed by increase in Ca/P ratio and the saturation of the solution with respect to CaCO_3_ [23]. However, the calcite formation might be also attributed to the effect of the particle size of BG [24] (as the mean particle size of the BG glass synthesized in alumina crucible was about twice smaller than of the BG glass prepared in Pt crucible [9]).

One important and intriguing result of the present study is the bioactivity of the BG-gly paste, which was higher than that of the neat BG. Despite the BG-gly sample also showing early calcite precipitation, at SBF exposure time longer than three days HA mineralization is observed, and after SBF exposure of seven days HA is the only crystalline phase present on the surface of BG.

**Figure 5:**
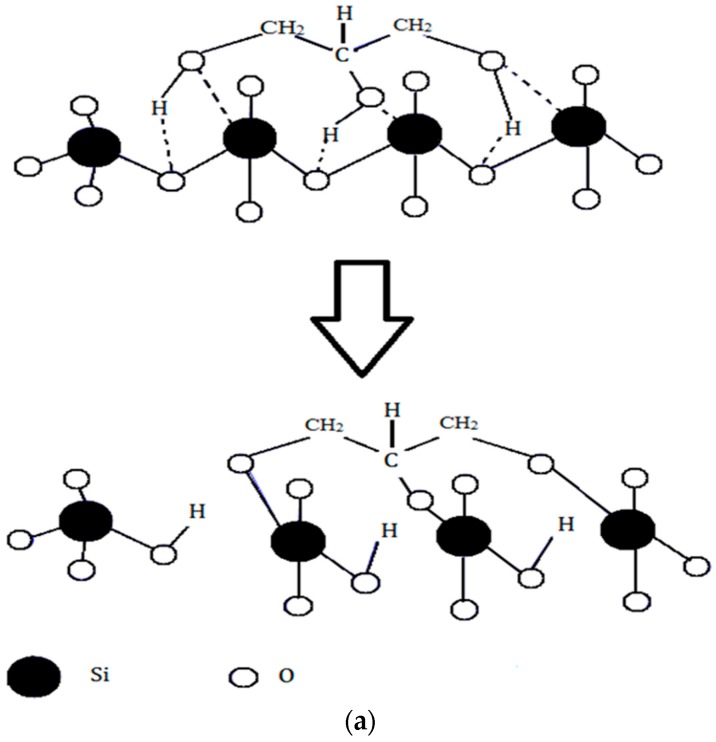

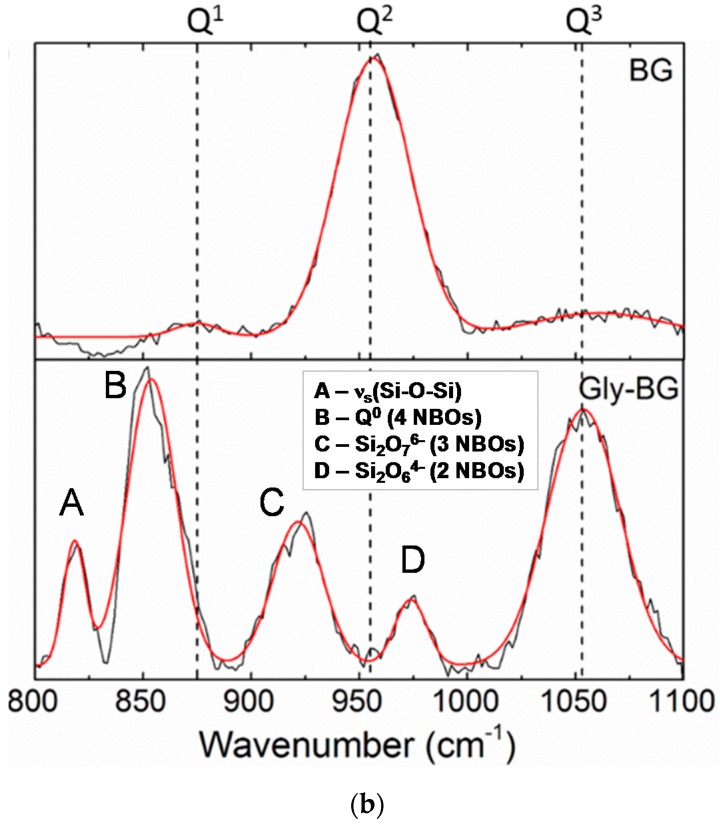
(**a**) Possible interactions of BG with glycerol leading to the decrease of the BG network connectivity and formation of Si–OH groups; (**b**) Raman spectra of BG (top) and BG-gly paste (bottom) [10].

The difference in the in vitro behavior between BG and BG-gly can be explained by structural features of the BG-gly paste. The results of our previous spectroscopic study on BG-gly [10] clearly indicate that glycerol chemically interacts with the surface of the BG particles. Typically, the reaction between gly and the BG surface can be described in a similar way as the reaction between siloxane bonds and alcohols, which was studied in the past by various researchers [25,26]. Thus, glycerol probably reacts with the BG surface to generate alkoxy-like, as well as silanol groups (Figure 5a). Assuming a Q^2^ site at the surface reacting with one molecule of gly, one can expect that through the alcoholysis of a Si-O bond a Q^3^ site is created in addition to a silanol group. This is supported, for example, by Raman spectroscopic data of the BG-gly paste (Figure 5b), which show significant differences between the BG-gly glass network and that of BG. Thus, the BG network consists predominantly of Q^2^ sites (absorption band at ca. 950 cm^−1^ [10]) in addition to small amounts of Q^1^ (ca. 875 cm^−1^) and Q^3^ (ca. 1050 cm^−1^) sites. Unlike BG, the network in BG-gly shows the band at 1050 cm^−1^ assigned to Q^3^ species being significantly enhanced as compared to the as-prepared BG [10]. Moreover, other intensive absorption bands at ca. 975 cm ^−1^ (Si_2_O_6_^4−^ units, possessing two non-bridging oxygens, NBOs), 925 cm^−1^ (Si_2_O_7_^6−^ units with three NBOs), 875 cm^−1^ (Q^0^, i.e., four NBOs) and ~825 cm^−1^ (symmetric ≡Si–O–Si≡ stretching vibration) [27,28] have been observed; whereas the band assigned to the Q^2^ units of BG disappeared upon interaction between BG and glycerol (Figure 5b). This indicates that the interaction of gly with BG leads to a significantly less polymerized glass network (at least at the glass surface) and to an increased number of silanol groups. As an obvious consequence, the bioactivity of the BG-gly sample is significantly improved as compared to that of the neat BG. This may also explain the faster leaching kinetics of Ca^2+^ and Mg^+2^ in BG-gly, as compared to the neat BG. 

Considering the ion release of the samples upon exposure to SBF, as well as their mineralization behavior, it is very intriguing that the BG sample mineralizes calcite together with hydroxyapatite; whereas the BG-gly sample shows the intermediate precipitation of calcite, which is then consumed/dissolved and, after 172 h of immersion in SBF, only HA is present on the surface of BG-gly. Firstly, the precipitation of calcite is somehow surprising, as the composition of the simulated body fluid indicates supersaturation conditions with respect to HA and, in fact, undersaturation conditions concerning calcite. However, it was shown in various studies that a strong release of Ca^2+^ from the bioactive glasses during the SBF test correlated with an abrupt PO_4_^3−^ depletion may create supersaturation conditions for calcite [23,24,29,30,31]. This may be the explanation also in the present case, as supported by the evolution of the ion concentrations of PO_4_^3−^ and Ca^2+^ (Figure 2c,d, respectively). It is shown that the concentration of Ca^2+^ in SBF strongly increases within the first 72 h for both samples, BG and BG-gly, that is, from 2.7 mmol/L to 3.6 and 3.9 mmol/L, respectively (Figure 2c); at the same time, PO_4_^3−^ ion concentration significantly decreases from 1 mmol/L to 0.22 and 0.21 mmol/L, respectively. Thus, it seems that the ion release behavior of both samples leads to favorable saturation conditions for calcite, which is observed to mineralize on their surface. Even more intriguing is the fact that BG-gly exhibits only HA on its surface after 168 h of immersion in SBF (Figure 3b), whereas in the case of BG sample, calcite and HA coexist on its surface (Figure 3a). The coexistence of calcite and HA on the surface of bioactive glasses exposed to SBF was reported in several studies. It was stated that calcite and HA co-precipitate in the very early stage of SBF immersion for glasses exhibiting high Ca^2+^ release ability [24], and thus both phases are present on their surface upon SBF exposure. This was shown for various bioactive glasses [24,29,31] as well as for our BG sample in the present study. However, the BG-gly system showed the disappearance of calcite and the presence of only HA upon 168 h of SBF immersion. This is a rather intriguing behavior which has not been reported so far. Various studies in the literature reported on the conversion of calcium carbonate (aragonite or calcite) into hydroxyapatite, for example, [32,33]. However, this process was typically carried out under hydrothermal conditions, namely, temperatures of ca. 150–250 °C and pressures up to 100 MPa [33]. Nevertheless, some studies can be found in literature reporting on the conversion of calcium carbonate into hydroxyapatite at room temperature in phosphate buffer solutions [34,35]. The conversion of calcium carbonate into HA is believed to rely mainly on the Ca ion release from the carbonate as well as on the presence of (hydrogen) phosphate ions in the solution. Moreover, the pH value of the solution seems to play a significant role: in a case study, it was shown that HA is generated from aragonite at pH values in the range from 7.4 to 8.0, whereas a lower pH values other calcium phosphate phases were generated [35].

In the present case, it is intriguing that BG shows the coexistence of calcite and HA, whereas BG-gly shows only the presence of HA on its surface. Considering the evolution of the pH of the SBF solution for both samples, it can be concluded that, beside the first few hours of exposure, both solutions show similar pH values. Thus, the difference in the surface mineralization of the samples can rely only on differences with respect to the ion release behavior. Indeed, as shown in Figure 2c, there is a clear difference between BG and BG-gly with respect to the release of PO_4_^3−^: the concentration of PO_4_^3−^ in the SBF solution of BG-gly decreases continuously from 3 h to 168 h of exposure to SBF test, indicating a continuous deposition of phosphate-containing phases on the surface of BG. On the other hand, in the case of the BG sample, the concentration of PO_4_^3−^ in the SBF solution drastically increases at exposure time higher than 72 h, indicating that the crystallization of phosphate phases on the surface of BG is suppressed.

Based on the obtained results, we assume that both samples, that is, BG and BG-gly, mineralize calcite on their surface in the early stage of their SBF exposure due to the strong release of Ca^2+^ and PO_4_^3−^ which probably creates (super)saturation conditions for calcite. At exposure times longer than 72 h, the BG and BG-gly behavior is different. Thus, the sample BG shows the coexistence of HA and calcite after 168 h of soaking in SBF; this can be explained by the conversion of calcite into HA, which is a dissolution and re-precipitation process [34]. The evolution of the PO_4_^3−^ concentration in the SBF solution from 72 h to 168 h of exposure time indicate that probably two concurrent processes occur, that is, PO_4_^3−^ dissolution from the glass and phosphate phases/HA precipitation, the first being dominating. Unlike in BG, the phosphate depletion in the SBF solution of BG-gly is continuous over the time; consequently, the precipitation of phosphate phases/HA on the surface of BG-gly seems to be dominating and overlaps the dissolution of phosphate from the glass. It seems that the modification of the BG surface with glycerol induces the difference in its mineralization behavior, as discussed above. However, more detailed investigation is needed in order to elucidate the precise reason of the selective HA mineralization on the surface of BG-gly.

## 5. Conclusions

In the present study, the bioactivity of a melt glass prepared in alumina crucible (BG), as well as of a BG-glycerol paste, was investigated. The obtained results allow following conclusions:

(i) the presence of small amount of aluminum in BG (~1.4 wt %, due to the preparation in an alumina crucible) suppresses the HA mineralization during the SBF test; this is most probably due to an increase of the network connectivity of the glass (i.e., Al acting as network former) and the enrichment of Al–OH groups at the surface of BG during SBF exposure;
(ii) the BG-gly paste shows an improved bioactivity as compared to the neat BG. This fact relies on chemical interaction of gly with the surface of BG, which results in a strong decrease of the network connectivity of BG (as shown by Raman spectroscopy), as well as a significant increase of the amount of surface silanol groups.

It is considered that the interaction between bioactive glass materials and organic carriers (e.g., polyalcohols) can be used to address specific biomedical applications which need injectable material formulations and, at the same time, to adjust/improve the intrinsic bioactivity of glasses upon altering their network connectivity and reactive surface groups such as silanols.
